# Targeting RIPK1 to modulate cell death and tumour microenvironment in cancer therapy

**DOI:** 10.1080/14756366.2025.2593800

**Published:** 2025-12-03

**Authors:** Jing Chen, Shijie Mao, Lingling Huang, Tao Zhu, Yun Bei

**Affiliations:** ^a^ Department of Pharmacy, Huzhou Central Hospital, Fifth School of Clinical Medicine of Zhejiang Chinese Medical University, Huzhou, Zhejiang, China; ^b^ Department of Pharmacy, Affiliated Huzhou Hospital, Zhejiang University School of Medicine, Huzhou, Zhejiang, China; ^c^ Department of Pharmacy, Huzhou Third Municipal Hospital Affiliated to Huzhou University, Huzhou, Zhejiang, China; ^d^ Department of Pharmacy, The Second Affiliated Hospital, Zhejiang University School of Medicine, Hangzhou, China

**Keywords:** Cancer, necroptosis, RIPK1, tumour metastasis, tumour microenvironment

## Abstract

Receptor-interacting serine/threonine-protein kinase 1 (RIPK1) is crucial in regulating inflammation, apoptosis, and necroptosis. Accumulating evidence highlights RIPK1 as a promising therapeutic target for various human diseases, including neurodegenerative disorders, autoimmune diseases, and cancer. In tumour cells, RIPK1 suppresses immunogenic cell death, promotes an immunosuppressive tumour microenvironment, which facilitates immune evasion, metastatic progression, and therapeutic resistance, contributing to an immunologically cold tumour phenotype. Therefore, targeting RIPK1 represents a promising therapeutic approach to overcome immune checkpoint blockade resistance and convert tumours into an immunologically hot phenotype. In this review, we summarise the biological functions of RIPK1 and elaborate on its roles in cancer progression in terms of the tumour immune microenvironment, tumour metastasis, and chemoresistance. Furthermore, we enumerate several identified RIPK1-targeted inhibitors with potential for cancer therapy. Although RIPK1 has been proposed as a potential anticancer target, there are still great opportunities and challenges that require further investigation.

## GRAPHICAL ABSTRACT

**Figure:**
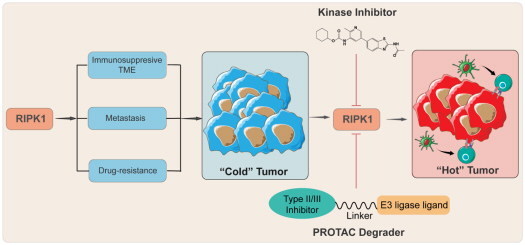

## 1. Introduction

Cell death is a critical process maintaining organismal development and adult homeostasis, which can be non-lytic and largely immunologically silent (apoptosis), or lytic and proinflammatory (necrosis).^1^ Elimination of excess cells during development is important to ensure normal morphogenesis and organogenesis; while in adult life, elimination of autoreactive immune cells, cancer cells, and damaged cells is essential to maintain the homeostasis.^2^ Cell death is involved in a variety of human diseases, such as neurodegenerative diseases and cancer, the former involves the death of neurons and the latter involves the failure to eliminate cells carrying cancerous mutations, which is one of the hallmarks of cancer.^3^ Different types of cell death can be classified based on morphology. For instance, apoptosis and necrosis are distinguished by their specific morphological features.^4^

Apoptosis, also known as programmed cell death (PCD), is a fundamental defense mechanism employed by the immune system to eliminate infected or damaged cells, which can be activated through endogenous and exogenous pathways.^5^ In endogenous apoptosis, also known as apoptosis *via* the mitochondrial pathway, cells sense intracellular stressors (i.e., growth factors or nutrient deprivation, DNA damage, and endoplasmic reticulum [ER] stress) and initiate self-destruction through the processes involving mitochondrial outer membrane permeability (MOMP).^6^ In extrinsic apoptosis, extracellular signals are recognised by death receptors on the cell membrane triggering cell death.^7^ These two pathways converge in the activation of caspase, a group of cysteine proteases.^8^ The caspase-3, -6, and -7 are effector caspases, these caspases cleave hundreds of intracellular substrate proteins, leading to the fragmentation of the cell into apoptotic bodies and ultimately resulting in cell death.^9^ The caspase-9 in the endogenous pathway and caspase-8 and -10 in the exogenous pathway, are promoter caspases that activate effector caspases. The last group of caspases (caspase-1, -4, -5, and -11) are classified as inflammatory caspases and induce an inflammatory PCD called pyroptosis. Apoptosis not only plays an important role in the pathogenesis of tumours, but also significantly impacts the effectiveness of tumour therapy.^10^^,^^11^

The induction of immunogenic cell death (ICD), including necroptosis and pyroptosis, represents a promising strategy to overcome apoptosis resistance and reactivate antitumor immunity in the tumour microenvironment (TME).^12^ By releasing damage-associated molecular patterns (DAMPs), ICD licences dendritic cell maturation and promotes cytotoxic T cell infiltration, thereby restoring immunosurveillance in the TME and converting immunologically cold to hot tumours.^13^^,^^14^ Clinical evidence has confirmed that patients with immunologically cold TME exhibit deficient cytotoxic T lymphocyte infiltration, primary resistance to immune checkpoint inhibitors, and significantly reduced overall survival.^15–17^ Therefore, reprogramming TME by inducing ICD holds transformative potential for enhancing immunotherapy efficacy.

Emerging evidence indicates that receptor-interacting serine/threonine-protein kinase 1 (RIPK1) as a critical stress sentinel that functions as a molecular switch governing cell survival, inflammatory responses, and ICD signalling.^18^^,^^19^ Through dynamic transitions between its scaffolding and kinase activities, RIPK1 orchestrates cell fate decisions by modulating survival versus death pathways and determining modes of cell death, ultimately shaping ICD-mediated remodelling of TME.^20^ RIPK1 was significantly upregulated in tumour cells of immune checkpoint blockade (ICB)-resistant murine models, where it drives TME immunosuppression by recruiting suppressive myeloid cells and impairing cytotoxic lymphocyte infiltration.^21^ The cancer-associated dysregulation of RIPK1 exhibits tumour type-specificity, hijacking survival and death related signalling networks to accelerate tumour progression.^22^^,^^23^ Alterations at the genetic, epigenetic and expression levels collectively enable RIPK1 to promote tumour metastasis, immune evasion, and therapy resistance.^23^ Of note, RIPK1 expression exhibits tumour type-specific heterogeneity, consequently enabling context-dependent protumor or antitumor functions dictated by distinct cancer lineages and cellular milieus.^23^^,^^24^ Despite these research advancements, the mechanistic basis for RIPK1’s paradoxical roles remain poorly defined, particularly how tumour-intrinsic factors (e.g., oncogenic drivers) and extrinsic cues (e.g., cytokine milieu) dictate its functional dichotomy.

While several excellent reviews have comprehensively delineated the fundamental biology of RIPK1 in cell death and inflammation, this review mainly focuses on its newly emerging and critical role as a pivotal regulator of the immunologically cold TME. In this review, we present a dedicated and in-depth discussion on how RIPK1 signalling actively suppresses ICD and promotes an immunosuppressive milieu, thereby driving resistance to ICB and other immunotherapies. Furthermore, we also summarise the most recent advances in targeting RIPK1, particularly through novel degradation strategies like proteolysis-targeting chimaeras (PROTACs), and their potential to overcome ICB resistance by reprogramming the TME, offering a timely perspective on translational and combination therapy strategies.

## 2. Structural domains and dynamic regulation of RIPK1

**Figure 1.**
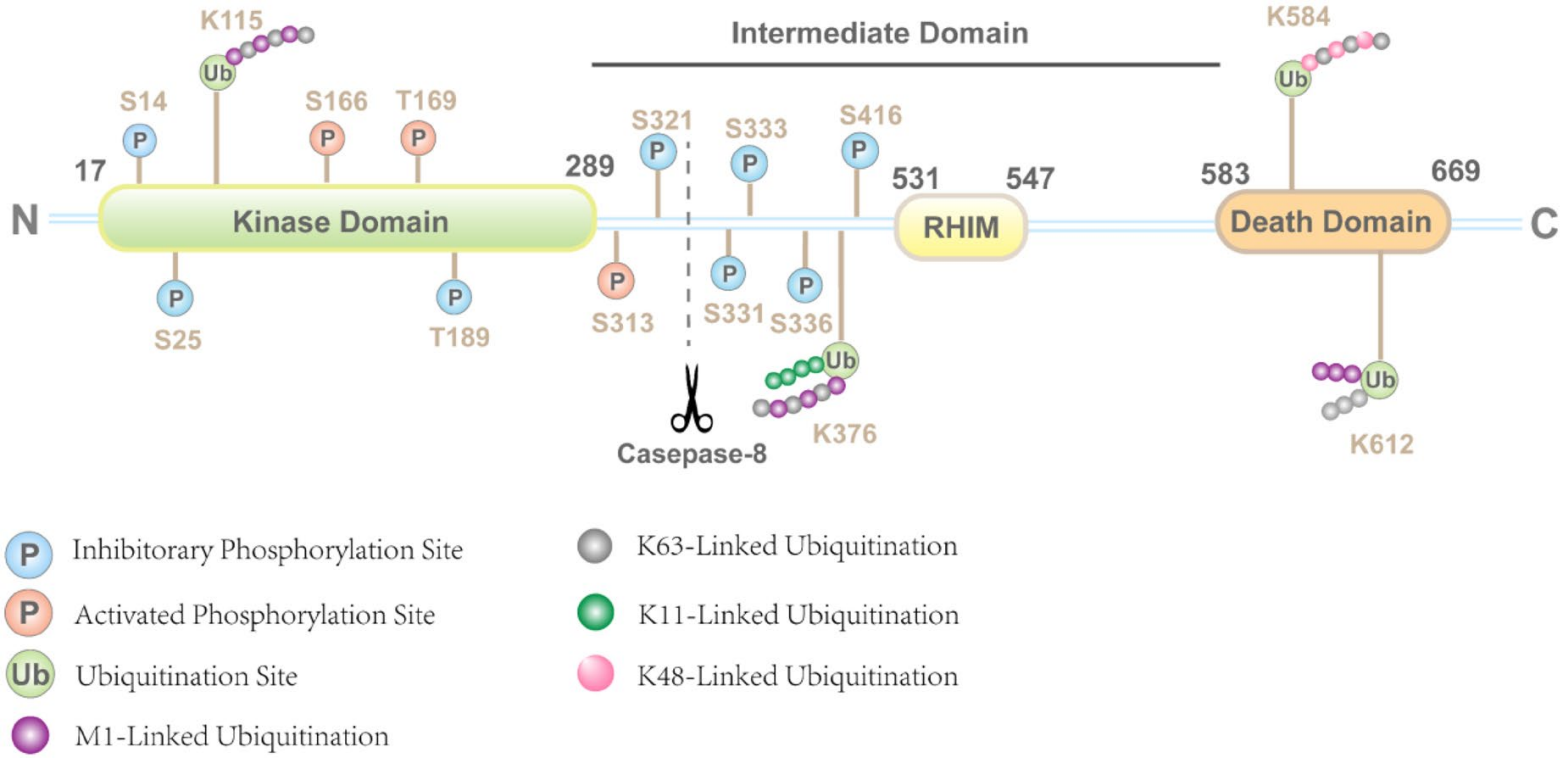
The domains and post-translational modifications discovered in human RIPK1. The structure of receptor-interacting serine/threonine-protein kinase 1 (RIPK1) consists of an N-terminal kinase domain (KD), a C-terminal death domain (DD) and a bridging intermediate domain (ID), which including a RIP homotypic interaction motif (RHIM). The cleavage of RIPK1 by caspase-8 inhibits RIPK1 activation by separating its KD from the DD. Phosphorylation at key residues directly modulates RIPK1 kinase activity (e.g., inhibitory phosphorylation at Ser25, activating autophosphorylation at Ser166), while diverse ubiquitin chain linkages dictate its stability, complex association, and signalling outcomes, mediated by specific E3 ligases (cIAPs, TRAFs, LUBAC) and deubiquitinating enzymes (A20, CYLD, OTUDs). Several identified phosphorylation site and ubiquitination site were also expressed in the schema. (This figure is created by the authors using Adobe Illustrator cc 2018.)

RIPK1 compose of three major domains: an N-terminal kinase domain (KD), an intermediate domain (ID) containing a RIP homotypic interaction motif (RHIM), and a C-terminal death domain (DD) (Figure 1). The KD of RIPK1 exhibits the typical serine/threonine kinase fold structure, including the N-lobe, C-lobe and the activation loop. A hydrophobic pocket located between the N-lobe and C-lobe allosterically regulates kinase activity.^25^ Phosphorylation of specific residues is critical for KD function: Gly45 and Lys47 facilitate ATP binding; Ser166 phosphorylation activates the kinase;^26^ phosphorylation at Ser25 inhibits catalytic activity^27^; and Ser320/Thr321 phosphorylation influences kinase activity and substrate binding.^28^ The DD typically comprises 80–100 amino acids, forms a fascicle-like structure consisting of six α-helices, enabling homologous or heterologous interactions. The C-terminal DD of RIPK1 primarily mediates homodimerization and heterodimerization with other DD-containing proteins, such as FADD, TNFR1, and Fas.^29^

The activity and functional outcomes of RIPK1 are dynamically controlled through a tightly interwoven network of mechanisms, including post-translational modifications (PTMs), integration into distinct signalling complexes, and interactions with specific regulatory factors. Phosphorylation and diverse ubiquitin chain linkages critically regulate its stability, complex association, and signalling output^26^^,^^27^^,^^30–33^ (Figure 1). Furthermore, lipid modifications such as S-palmitoylation modulate its kinase activation, subcellular localisation, and ability to engage downstream partners.^34^

Crucially, RIPK1’s role is defined by its dynamic incorporation into specific macromolecular complexes: within ubiquitin-decorated Complex I, RIPK1 acts as a scaffold facilitating NF-κB/MAPK activation and cell survival^30^^,^^32^^,^^35^^,^^36^; upon Complex I disassembly and inhibition of pro-survival signalling, RIPK1 can form cytosolic Complex IIa *via* its death domain interaction with FADD, promoting caspase-8 activation and RIPK1-mediated apoptosis (RDA)^30^^,^^35^^,^^37^; conversely, under caspase inhibition, RIPK1 kinase activity drives necrosome (Complex IIb) formation *via* RHIM-mediated recruitment of RIPK3 and MLKL, executing necroptosis.^35^^,^^38^^,^^39^ Moreover, regulatory kinases (TAK1, TBK1, IKKs, MK2) phosphorylate RIPK1 to restrain its pro-death kinase activation.^32^^,^^40–43^ The precise interplay between PTMs, complex dynamics, and regulatory factors constitutes a sophisticated control network that dictates whether RIPK1 promotes survival/inflammation or drives cell death in response to diverse stimuli.^31^^,^^40^

## 3. Roles of RIPK1 in cell death

### 3.1. RIPK1 affects inflammation and cell survival

As a scaffold protein, RIPK1 regulates cellular inflammation and survival by forming complexes with different aptamers without triggering kinase activity. Current studies suggest that TNF-α binds to TNFR1, promoting trimerization and recruiting two death domain-containing proteins (adaptor proteins TRADD and RIPK1) at the DD of TNFR1 to form complex I.^36^ TRADD stabilises RIPK1’s function as a scaffold protein by recruiting various ubiquitin ligases to ubiquitinate complex I, thereby inhibiting KD phosphorylation and activation. Previous studies have identified multiple E3 ubiquitin ligases, such as TRAF2/5, cIAP1/2 and LUBAC, which stabilise complex I through ubiquitination.^30^^,^^32^ This modification prevents RIPK1 dissociation from complex I or enhances its anchoring effect, limiting its transition into complex II and subsequent cell death.^44^ When complex I undergoes deubiquitination, the ubiquitin chain is removed, KD activity is restored, and complex I dissociates. Additionally, certain key amino acid residues are crucial for complex I formation. For instance, mutation of the RIPK1 ubiquitination site K376 reduces K11-, K63-linked, and linear ubiquitination, promoting the assembly of death-inducing signalling complexes.^45^ Furthermore, several kinases, including MK2, TAK1, TBK1/IKKε, and JAK1, have been found to phosphorylate RIPK1, inhibiting its kinase activity and restricting its cytosolic activation and subsequent integration into complex II.^32^^,^^41–43^ In complex I, RIPK1 functions solely as a scaffold protein without kinase activity, potentially due to conformational changes in KD. There are two possible reasons for this: Firstly, the binding of the DD to TRADD induces a conformational change in KD, thereby restricting autophosphorylation. Secondly, K63-linked ubiquitination mediated by TRAF2/5 and cIAPs alters the conformation of KD, which requires deubiquitinase activity to reverse the conformational change.

Accumulating evidence indicates that RIPK1 promotes intracellular survival and inflammatory responses primarily *via* the activation of the NF-κB signalling pathway.^46^^,^^47^ Specifically, the K63-linked ubiquitin chain on RIPK1 recruits TAB2/3 and TAK1, followed by LUBAC-mediated linear ubiquitination of M1 to recruit the NEMO-IκB kinase (IKK) complex. This recruitment activates both the NF-κB and MAPK pathways, thereby enhancing the transcription of pro-inflammatory and pro-survival genes such as Bcl2, A20, and c-FLIP.^35^ Research has demonstrated that the loss of RIPK1 scaffold function may inhibit NF-κB signalling, thereby triggering epithelial cell apoptosis.^48^ In immune cells, deletion or mutation of the deubiquitinating enzyme OTUD1 leads to excessive K63-linked ubiquitination at lysine 627 of RIPK1, resulting in hyperactivation of the NF-κB signalling pathway and contributing to intestinal inflammation.^33^ During early atherosclerotic lesions, RIPK1 predominantly drives NF-κB-dependent inflammation, and its knockdown reduces inflammatory cell activation, thus preventing the progression of atherosclerosis.^49^ Notably, while the kinase activity of RIPK1 is critical for its role in cell death, it is dispensable for its pro-survival NF-κB/MAPK signalling function.

### 3.2. RIPK1 mediates apoptosis

**Figure 2.**
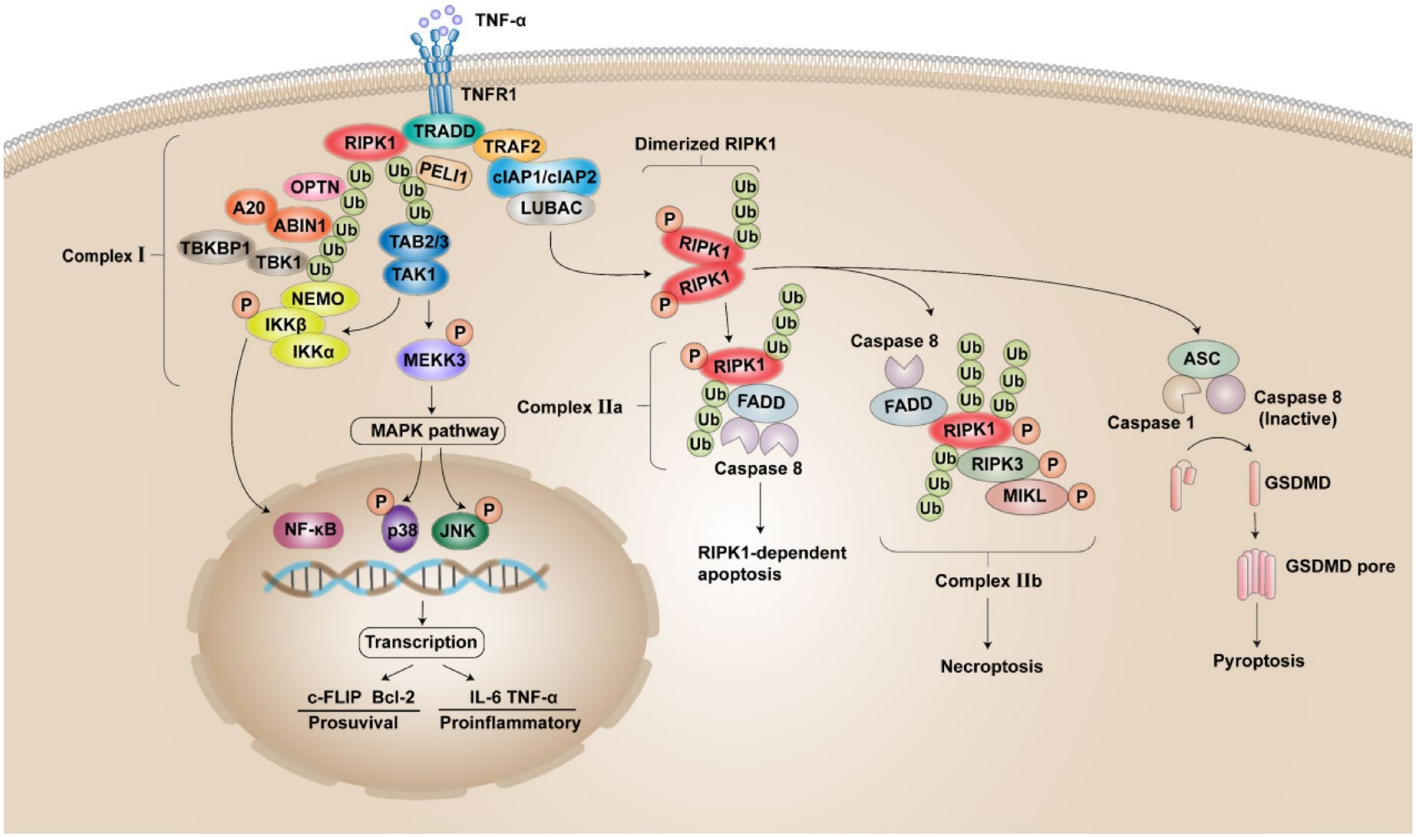
Core mechanisms of cell fate decisions mediated by the TNF-α signalling pathway. 1) RIPK1-mediated pro-survival and pro-inflammatory pathway: TNF-α binding to tumour necrosis factor receptor 1 (TNFR1) triggers receptor trimerization and recruits the death domain (DD)-containing adaptor proteins TRADD and receptor-interacting serine/threonine-protein kinase 1 (RIPK1) to form Complex I. TRADD recruits E3 ubiquitin ligases (e.g., TRAF2/5, cIAP1/2, LUBAC), which mediate K63-linked polyubiquitination of RIPK1. This ubiquitin scaffold recruits and activates downstream kinases (TAB2/3–TAK1 complex), ultimately leading to activation of the NF-κB and MAPK pathways. M1-linked ubiquitin chains on RIPK1 recruit the NEMO-IκB kinase (IKK) complex, TBK1, A20, ABIN1 (A20-binding inhibitor of NF-κB-1), and OPTN. Activation of TAK1 and IKK facilitates NF-κB signalling, driving transcription of pro-inflammatory and pro-survival genes. 2) RIPK1-dependent apoptosis (RDA): Upon RIPK1 deubiquitination or insufficient ubiquitination, its DD directly engages Fas-associated death domain protein (FADD). This interaction facilitates procaspase-8 recruitment, leading to assembly of Complex IIa (RIPK1-FADD-procaspase-8) that executes RDA. 3) Necroptosis: Suppression of caspase-8 activity enables kinase-active RIPK1 to recruit RIPK3 and catalyse its phosphorylation. RIPK3 subsequently phosphorylates mixed lineage kinase domain-like pseudokinase (MLKL), triggering necrosome (Complex IIb) formation. This induces MLKL oligomerisation, plasma membrane permeabilization, and necrosis execution. 4) Pyroptosis: RIPK1 kinase activity promotes oligomerisation of inactive caspase-8, thereby inhibiting caspase-8-mediated apoptosis. This permits cells to undergo alternative death pathways such as necroptosis or pyroptosis. Concurrently, RIPK1 directly promotes NLRP3 inflammasome assembly to recruit ASC, leading to caspase-1 activation, GSDMD cleavage, and ultimately pyroptosis execution. (This figure is created by the authors using Adobe Illustrator cc 2018.)

Although the DD of RIPK1 is crucial for stabilising complex I and ensuring cell survival, it also plays an essential role in apoptosis. When pro-survival signalling through complex I is disrupted, deubiquitylation of RIPK1 following cIAPs deletion, RIPK1 dissociates from TNFR1; subsequently, its inhibitory phosphorylation and ubiquitination are removed, enabling the DD to bind FADD and form cytoplasmic complex IIa (Figure 2).^37^^,^^50^ This interaction recruits and activates caspase-8, initiating the downstream caspase cascade that leads to RDA.^35^ Is the occurrence of RDA independent of RIPK1’s kinase activity? Studies have demonstrated that blocking Ser 321 inhibitory phosphorylation of RIPK1 enhances its interaction with FADD during activation, thereby sensitising cells to TNFα-induced RDA.^30^ Additionally, TBK1 has been identified as an endogenous inhibitor of RIPK1, and embryonic lethality in TBK1^-/-^ mice depends on RDA mediated by RIPK1 activation upon TBK1 deletion.^31^ Unlike necrosis, RDA is mediated by complex IIa activated by RIPK1 rather than RIPK3. This may be attributed to caspase-8 cleaving RIPK1 upon activation, which removes the DD and RHIM domains, preventing RIPK1 from recruiting RIPK3 and FADD; thereby, blocks the amplification of apoptotic signals and inhibits necroptosis. Besides, a recent study also demonstrated that RIPK1 restrictsed ZBP1- and TRIF-mediated RIPK3 activation in a DD-dependent manner, thus inhibiting necroptosis.^51^ From a molecular structural perspective, this suggests that DD-mediated binding to complex IIa may induce conformational changes in the RIPK1 middle domain, thereby rendering its RHIM inaccessible for recruiting and activating RIPK3, which ultimately suppresses necrosome assembly.

### 3.3. RIPK1 mediates necroptosis

RIPK1 kinase-dependent necroptosis serve as the primary executor of cell death in response to extracellular inflammatory signalling. The crucial function of the KD of RIPK1 is to trigger necroptosis. When the apoptotic pathway is inhibited (e.g., caspase-8 inactivation or insufficiency), RIPK1 cannot be cleaved, and its C-terminal DD promotes RIPK1 dimerisation, leading to its activation. Activated RIPK1 binds to RIPK3 through the RHIM in its intermediate domain, subsequently activating RIPK3, which in turn activates mixed lineage kinase domain-like protein (MLKL) (Figure 2).^38^ Necrosomes (complex IIb), composed of RIPK1, FADD, caspase-8, RIPK3 and MLKL, are formed to execute necroptosis.^35^ Phosphorylated MLKL directly binds to phosphatidylinositol phosphate (PIP) and diphosphatidylglycerol in the plasma membrane or organelle membrane, leading to membrane disruption and cell lysis.^38^ Caspase inhibition is a prerequisite for susceptibility to necroptosis. Caspase-8, as the most important caspase regulating necroptosis, inhibits necroptosis by cleaving RIPK1, separating the N-terminal KD from the C-terminal part of the molecule, thereby preventing RIPK1 kinase activation by dimerisation of the C-terminal DD.^52^^,^^53^ Research has demonstrated that a mutation at the caspase-8 cleavage site (Asp325) in RIPK1 prevents its cleavage, accelerating cell death.^53^ Knock-in mice expressing catalytically inactivated caspase-8 (C362A) exhibited embryonic lethality due to MLKL-dependent necroptosis, a phenotype consistent with that observed in caspase-8-deficient mice.^30^ This may result from the decreased formation of complex IIa and the gradual shift of RIPK1 towards complex IIb in cells, consequently promoting necroptosis. The kinase activity of RIPK1 is essential for both RDA and necroptosis, and autophosphorylation of Ser166 of its KD has been widely used as a marker of RIPK1 activation.^39^ RIPK3 comprises an N-terminal kinase domain and a C-terminal intermediate domain that contains a RHIM domain that mediates binding to RIPK1. Studies have demonstrated that perinatal lethality in mice caused by the RHIM mutation of RIPK1 is due to necroptosis mediated by the ZBP1-RIPK3-MLKL pathway and apoptosis mediated by RIPK1.^54^ In normal conditions, activated RIPK1 competitively binds to RIPK3 *via* RHIM to prevent ZBP1 from initiating RIPK3/MLKL-dependent necroptosis.^39^

### 3.4. RIPK1 mediates autophagy

Autophagy, a cellular survival mechanism under metabolic stressors (nutrient deprivation, hypoxia, oxidative stress, or pathogen infection), is mediated by double-membrane autophagosomes that sequesters and degrade damaged components. RIPK1, a multifunctional signalling hub, bidirectionally regulates autophagic flux *via* metabolic reprogramming and kinase cascades. During starvation stress, RIPK1 deficiency leads to aspartate accumulation, AMPK inhibition, and impaired autophagy, whereas it promotes basal autophagy under normal conditions.^55^ This duality is supported by another study showing that RIPK1 negatively regulates TFEB (a master autophagy transcription factor) through ERK-mediated phosphorylation, reducing basal autophagic flux independently of its kinase activity.^56^ Thus, RIPK1’s dual regulation of autophagy reflects distinct mechanisms under normal growth and starvation conditions. Notably, in cancer research, RIPK1 upregulation in melanoma cells under endoplasmic reticulum stress induces autophagic pathways to clear damaged organelles, enhancing cancer cell survival.^57^ This suggests that inhibiting RIPK1 in tumour cells may induce autophagic defects, increase apoptotic sensitivity, and emerge as a novel strategy to overcome autophagy-dependent chemoresistance in cancer.

### 3.5. Potential links between RIPK1 and other cell death modalities

Pyroptosis is a form of PCD mediated by gasdermin (GSDM) family proteins (e.g., GSDMD), characterised by plasma membrane pore formation and intense inflammatory responses. RIPK1 engages in significant crosstalk with pyroptotic pathways. Inhibition of RIPK1 kinase activity reduces caspase-8 and cleaved GSDMD expression in cardiac endothelial cells, thereby attenuating pyroptosis.^58^ Notably, RIPK1 and the pyroptosis execution protein GSDMD share upstream regulatory mechanisms. Palmitoylation serves as a broad-spectrum initiator of PCD: it facilitates GSDMD cleavage and pore formation during pyroptosis,^59^ while in RDA and necroptosis, DHHC5-mediated palmitoylation of RIPK1 (dependent on its K63 ubiquitination) enhances the hydrophobicity of its kinase domain, promoting homotypic interactions and trans-autophosphorylation, ultimately triggering cell death.^34^ This suggests that RIPK1 and GSDMD may converge on common upstream activation mechanisms, enabling context-dependent coordination of distinct death modalities. Additionally, RIPK1 indirectly influences pyroptosis by modulating inflammasome activation. Under specific stress conditions, RIPK1 may facilitate NLRP3 inflammasome assembly and activation *via* mitochondrial fission factor DRP1 and reactive oxygen species (ROS), thereby promoting caspase-1-mediated GSDMD cleavage and amplifying pyroptotic signalling and inflammatory cytokine release (e.g., IL-1β).^60^^,^^61^ Future research should elucidate how metabolic microenvironments (e.g., glucose deprivation or fatty acid accumulation) dynamically regulate coordinated palmitoylation of RIPK1 and GSDMD to determine the switch between pyroptosis and other cell death fates, offering precise therapeutic strategies for diseases involving metabolic-immune crosstalk.

Ferroptosis is an iron-dependent, regulated cell death modality driven by lipid peroxidation. Although direct evidence is still emerging, compelling indirect links and theoretical intersections exist between RIPK1 and ferroptosis. The core driver of ferroptosis, lipid peroxidation, is a process highly dependent on intracellular redox status and metabolic activity.^62^ RIPK1 activation can be induced or amplified by various metabolic stresses (e.g., ER stress) and ROS.^63^ Conversely, RIPK1 activation may facilitate or amplify ferroptosis by disrupting mitochondrial function, depleting antioxidants such as glutathione, or indirectly creating a pro-lipid-peroxidative microenvironment through pro-inflammatory signalling.^62^ A recent *in vivo* study demonstrated that pharmacological inhibition of RIPK1 activity markedly scavenged free radicals and suppressed lipid peroxidation, exerting a robust anti-ferroptotic effect.^64^ Although the precise molecular mechanisms require further elucidation, the close interplay among RIPK1, oxidative stress, and metabolic dysregulation suggests that RIPK1 may serve as a critical bridge linking death receptor signalling to ferroptosis. For instance, in TAK1-deficient models, RIPK1 activation triggers intense ROS bursts, a key execution event in ferroptosis. Thus, targeting RIPK1 may offer a novel therapeutic strategy for ferroptosis-related pathologies by modulating cellular redox homeostasis.

Although genetically engineered mouse models (e.g., *RIPK1* knockout or kinase-dead mutants) serve as indispensable tools for elucidating the dual roles of RIPK1 in cell death, inflammation, and autophagy, their inherent limitations necessitate cautious interpretation. For instance, systemic knockout strategies may exaggerate phenotypic outcomes and fail to fully recapitulate the spatial and context-specific regulation of RIPK1 in human diseases.

## 4. Dual molecular mechanisms of RIPK1 in cancer progression

The activity of RIPK1 plays a pivotal role in regulating cell survival and death pathways. Increasing evidence suggested that alterations in function within cancer cells profoundly influence tumour progression.^65^ Dysregulation of PCD pathways, such as ICB and disrupted apoptosis pathways, represents a hallmark of tumorigenesis. Cancer cells often acquire survival advantages through mutations that impair the normal PCD pathway, and evasion of PCD constitutes one of the critical mechanisms underlying cancer progression.^66^ Necroptosis, a form of fail-safe cell death occurring when apoptosis fails, exhibits potent immunogenicity and can activate immune cells to eliminate cancer cells, a process known as ICD.^65^ Besides, inflammatory responses triggered by necroptosis may paradoxically promote tumour development by inducing genomic instability, angiogenesis, enhanced cell proliferation, and accelerated metastasis.^67^ As a central regulator of cell death, RIPK1’s role in cancer progression remains context-dependent, with its dual functionality offering both pro-tumorigenic and anti-tumorigenic effects. Extensive studies indicate that RIPK1 can stabilise its scaffold function *via* PTMs, including inhibitory phosphorylation, ubiquitination, palmitoylation, and other modifications, thereby suppressing apoptosis and necroptosis pathways.^34^^,^^43^^,^^68^ Conversely, appropriate activation of RIPK1 activity serves as a favourable condition for necroptosis, potentially promoting ICD in cancer cells. Moreover, recent evidence also suggests that cancer cells hijack RIPK1 (i.e., stabilise its scaffold function) to enhance cell survival, induce TNF insensitivity, and confer resistance to immunotherapy.^69^

### 4.1. RIPK1 expression varies in different types of cancers

To date, studies on various types of cancer have demonstrated distinct levels of RIPK1 expression in cancer cells or tissues. The majority of research indicates that RIPK1 overexpression contributes to cancer progression (Table 1). In lung cancer research, RIPK1 has been found to display significantly elevated expression levels in both human and mouse cancer tissues, as well as in various cancer cell lines.^70^ Additionally, a recent study revealed that reduced degradation of RIPK1 mediated by the OTUD6B deubiquitinase significantly accelerated the progression of lung cancer in patients.^71^ Overexpression of RIPK1 has also been observed in glioblastomas and is strongly correlated with poor prognosis. RIPK1 was shown to activate several pro-tumour genes (NF-κB and Akt) and suppress the expression of the tumour suppressor gene p53 in glioblastoma cells.^72^ High expression of RIPK1 has also been reported in pancreatic ductal adenocarcinoma.^73^

**Table 1.** The dual roles of RIPK1: oncogenic vs. tumour-suppressive functions across human cancers.

Cancer type	Model type	Expression of RIPK1	RIPK1 function	Mechanism and outcome	Ref.
Glioblastoma	Humanised	Upregulation	Protumoral	NF-κB/Akt activation ↑, Expression of p53 ↑ Prognosis ↓	^72^
Diffuse glioma	Humanised	Upregulation	Protumoral	Prognosis ↓, Survival ↓ Activation of TIME ↑	^112^
Gastric cancer	Humanised	Upregulation	Protumoral	Survival ↓, Invasion ↑	^113^
	Murine	Upregulation	Protumoral	Invasion induced by NF-κB/AP-1-VEGF-C signalling pathways ↑	^113^
NSCLC	Humanised	Downregulation	Antitumoral	Survival ↓	^114^
PDAC	Humanised	Upregulation	Antitumoral	Survival ↑	^115^
HNSCC	Humanised	Downregulation	Antitumoral	Protumorigenic properties ↑	^75^
Lymphoma	Humanised	Downregulation	Protumoral	Proliferation ↓	^76^
HCC	Humanised	Upregulation	Protumoral	AKT/Bcl-2/BAX Activation ↑, Survival ↓	^116^
CSCC	Humanised	Upregulation	Protumoral	Lymph node metastasis ↑ Survival ↓	^117^
Cervical cancer	Humanised	Upregulation	Protumoral	Regulation of NF-κB and TNF ↑, Migration ↑, Invasion ↑, Growth ↑, Prognosis ↓	^118^
Melanoma	Humanised	Upregulation	Protumoral	Activation of NF-κB ↑, Proliferation ↑, TNFα-induced apoptosis ↓	^119^
Breast cancer	Humanised	Upregulation	Protumoral	Tumour size and grade ↑, Expression of p53 ↓	^120^
TNBC	Humanised	Downregulation	Antitumoral	Survival ↓	^74^
COAD	Murine	Downregulation	Antitumoral	Resistance to Irinotecan ↑	^121^
RCC	Humanised	Upregulation	Protumoral	Tumour grade ↑, Necrotic features ↑	^122^

However, some studies indicate that RIPK1 levels are significantly reduced in several certain cancers (Table 1). For instance, RIPK1 depletion has been observed in the cancer tissue of patients with triple-negative breast cancer (TNBC) compared to normal breast tissue, and this downregulation is highly associated with improved patient survival. Conversely, RIPK1 levels are elevated in several other breast cancer subtypes.^74^ Similarly, in metastatic head and neck squamous cell carcinoma, downregulation of RIPK1 is possibly mediated by enhanced methylation of the RIPK1 promoter in tumour cells and enhances protumorigenic properties such as cell migration.^75^ Moreover, clinical studies have demonstrated that RIPK1 is significantly downregulated in tumour cells of lymphoma patients, thereby enhancing their proliferative capacity.^76^ Conventional bulk sequencing or tissue homogenate assays yield population-averaged signals, potentially obscuring the significant heterogeneity of RIPK1 expression across distinct tumour cell subpopulations (e.g., cancer stem cells, differentially differentiated tumour cells, and immune cells within the tumour microenvironment). Although phosphorylated MLKL (p-MLKL) detection *via* immunohistochemistry has been reported in various human cancers (e.g., colorectal and breast carcinomas) as a surrogate readout for necroptosis, its specificity and reliability remain challenged. Current interpretation of p-MLKL staining thus necessitates integration with morphological analysis (e.g., cellular swelling, membrane rupture) and complementary molecular markers (e.g., RIPK3 phosphorylation) for robust assessment.

### 4.2. Role of RIPK1 on the tumour microenvironment

**Figure 3.**
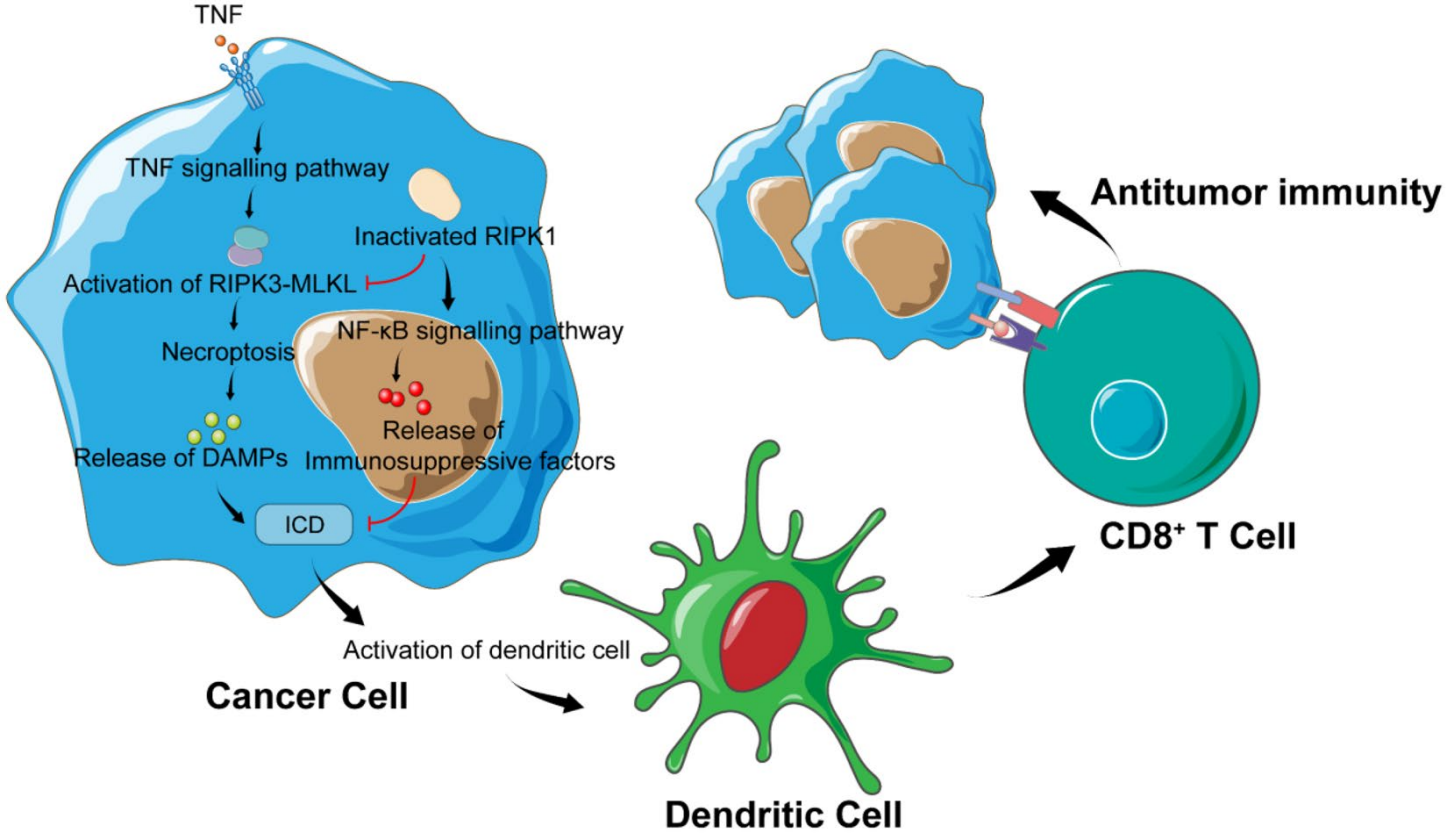
RIPK1 reprograms the tumour immune microenvironment by suppressing immunogenic cell death (ICD) through its scaffolding function. RIPK1 blocks RIPK3-MLKL oligomerisation to inhibit necroptosis and attenuates damage-associated molecular pattern (DAMP) release (e.g., HMGB1, ATP), thereby impairing dendritic cell activation. Concurrently, RIPK1 scaffolding sustains NF-κB activation to drive secretion of immunosuppressive cytokines (TGF-β, IL-10) and recruitment of inhibitory myeloid cells (M2-TAMs, MDSCs), collectively diminishing cytotoxic CD8^+^ T cell priming and antitumor immunity. (This figure is created by the authors using Adobe Illustrator cc 2018.)

Through its unique scaffold function, RIPK1 regulates immunosuppressive signals in multiple dimensions within the TME, thereby becoming a critical regulatory node for cancer immune escape (Figure 3). In its inactive state, RIPK1 functions as a scaffold protein that protects cancer cells from immunodetection and death effects.^69^ The stabilisation of RIPK1’s scaffold function facilitates the assembly of complex I and activates NF-κB signalling in the TNF pathway in cancer cells. By recruiting proteins such as TRADD, TRAF2, and TAK1, complex I activates the NF-κB signalling pathway, promoting the expression of anti-apoptotic genes (e.g., c-FLIP, Bcl-2 family members) and inhibiting caspase-8-mediated apoptosis. Persistent activation of NF-κB further induces the secretion of immunosuppressive cytokines (e.g., IL-6, TGF-β), shaping an immunosuppressive TME, enhancing cancer cell survival, and reducing the infiltration of T and NK cells expressing TNF superfamily ligands.^21^ Studies have demonstrated that inhibition of the NF-κB pathway promotes the transition from sublethal necrosis to lethal necroptosis, preventing the release of relevant cytokines and simultaneously inhibiting liver cancer development.^77^ This intriguing finding strongly suggests that this outcome is most likely caused by the inhibition of the NF-κB pathway and the subsequent activation of RIPK1/RIPK3 necrosomes after the loss of its scaffold function. Moreover, RIPK1’s scaffold function maintains an immunosuppressive TME by inhibiting the infiltration of FOXP3^+^ Treg cells and the secretion of pro-inflammatory factors (e.g., IFN-γ, IL-2). Following RIPK1 degradation and loss of scaffold function, cancer cells become more sensitive to therapy-induced TNF and interferon, thereby enhancing the immunostimulatory effects of radiotherapy and immunotherapy.^69^ Notably, the complete knockdown of RIPK1 in cancer cells results in profound alterations within the tumour microenvironment, characterised by increased infiltration of effector T cells, reduced presence of immunosuppressive myeloid cells, and augmented secretion of immune-stimulating cytokines.^21^^,^^78^ However, direct mechanistic evidence linking RIPK1 signalling to the functional modulation of myeloid-derived suppressor cells (MDSCs), Treg cells activity, and dendritic cell (DC) cross-priming remains scarce. Future investigations should employ PROTAC degraders (e.g., LD4172) or cell-specific genetic knockout models coupled with clinical immunomonitoring to directly validate the impact of RIPK1 ablation on MDSC/Treg/DC functionality, moving beyond inferences from murine cytokine data to establish precise targets for combinatorial immunotherapy.

Activation of cell necroptosis mediated by the enzymatic activity of intra-tumoral RIPK1 enhances tumour ICD. Inflammatory cytokines released by cells not undergoing programmed death facilitate the maturation and activation of immune cells, thereby mediating ICD in cancer cells.^79^ As a form of non-PCD, the introduction of necrotic apoptotic cells into the tumour microenvironment promotes anti-tumour immunity dependent on immune cell activation and increases the antigen load of tumour-associated antigen-presenting cells.^80^ Activation of the necrosome composed of RIPK1/RIPK3 is critical for phosphorylation of MLKL, which induces necroptosis. Studies have demonstrated that MLKL activation mediated by the RIPK1/RIPK3 complex enables membrane permeabilization, leading to the release of cellular contents, including DAMPs and cytokines/chemokines.^81^ Data from breast cancer research indicates that the formation of RIPK1/RIPK3 necrosomes is essential for mediating ICD in cancer cells, while autophagic degradation of necrosomes results in ICD failure in breast cancer cells.^82^ Clinical studies also reveal that RIPK1 and MLKL mRNA expression levels exhibit a significant downward trend in breast cancer tissues, potentially increasing intrinsic resistance to lethal signals and enabling evasion of immune surveillance.^83^ Additionally, TRAF6 has been shown to directly interact with RIPK1 *via* Lys48-linked polyubiquitination, reducing p-RIPK1 levels and inhibiting the activation of the RIPK1–RIPK3–MLKL axis in colorectal cancer cells, thereby suppressing necroptosis and promoting tumour progression.^84^ Interestingly, in a lung cancer study, mice deficient in RIPK1 kinase activity (*Ripk1^K45A/K45A^*) exhibited a 38.3% reduction in tumour nodules compared with wild-type mice, whereas no difference in the number of tumour nodules was observed in mice with RIPK3 kinase deficiency (*Ripk3^K51A/K51A^*).^85^ Unlike mice with complete RIPK1 deletion, T cells in *Ripk1^K45A/K45A^* mice lack RIPK1 kinase activity but retain their scaffold function, potentially leading to more stable peripheral T cells and enhancing the likelihood of ICD in tumour cells. Recent studies have demonstrated that RIPK1-null mice exhibit increased sensitivity of peripheral T cells to caspase-8 and TNFR1-mediated apoptosis, while *Ripk1^K45A/K45A^* mice maintain stable populations of naive CD4^+^ and CD8^+^ T cells, indicating that the scaffold function of RIPK1 is sufficient for peripheral T cell homeostasis independent of its kinase activity.^86^ However, previous studies reported that DAMPs released by dying cells (e.g., HMGB1, ATP) are insufficient on their own to activate CD8^+^ T cell cross-presentation and must rely on activation of the RIPK1-NF-κB signalling axis within dying cells, which is essential for enhancing the antigen presentation capacity of DC and activating T cells.^87^ Thus, the RIPK1-NF-κB signalling axis promotes immunosuppression in surviving cancer cells, whereas decoupling of dual RIPK1 signalling in dying cancer cells can trigger the immune microenvironment to transit from suppression to activation, mediating increased ICD in cancer cells.

However, it must be emphasised that these findings predominantly stem from genetically modified mouse models or specific culture conditions. Their direct applicability to the complex tumour microenvironment in humans, and whether RIPK1-driven necroptosis constitutes a widespread and critical cell death pathway in human cancers, remain to be thoroughly validated using more specific tools and clinically relevant models. Integrating single-cell RNA sequencing (scRNA-seq) with phosphoproteomics or spatial transcriptomics holds promise for simultaneously resolving RIPK1 expression, its activation status (e.g., phosphorylation levels), and its interactions with mutational drivers and immune infiltration patterns at single-cell resolution. This approach is crucial for elucidating the distinct roles of RIPK1 across different cellular subpopulations (e.g., tumour cells, immune cells) and for establishing predictive biomarkers.

### 4.3. Role of RIPK1 in tumour metastasis

Researchers have found that human and mouse tumour cells induce necroptosis in endothelial cells, which promotes tumour cell extravasation and metastasis. McCormick *et al.* reported that RIPK1 expression was down-regulated in head and neck squamous cell carcinoma, suggesting that downregulation of RIPK1 expression promoted by epigenetic changes during tumour progression allows tumour cells to escape anoikis, which may stimulate tumorigenesis by enhancing the metastatic capacity of tumour cells.^75^ Treatment of mice with a RIPK1 inhibitor or endothelial cell-specific deletion of RIPK3 significantly reduced tumour cell-induced endothelial necroptosis, tumour cell extravasation, and metastasis.^88^ Another study found that RIPK1 promoted lymph node metastasis of gallbladder cancer by stimulating the binding of NF-κB and AP-1 to the VEGF-C promoter and up-regulating the expression of VEGF-C.^89^ RIPK1 regulates tumour cell migration and invasion by interacting with proteins associated with the cytoskeleton, such as F-actin and vimentin. siRNA knockdown of RIPK1 resulted in growth and migration inhibition of NSCLC cells *in virtro.*^90^

### 4.4. Role of RIPK1 in chemoresistance

Genetic interaction screening demonstrated RIPK1 regulates the TNF signalling pathway *via* NF-κB in cancer cells and plays a critical role in modulating cell death.^91^ This regulation facilitates the production of immunosuppressive chemokines, thereby enhancing cancer cell survival and inhibiting the infiltration of T cells and NK cells expressing TNF superfamily ligands. Knockout of RIPK1 expression in cancer cells impaired chemokine secretion, reduced the recruitment of ARG1^+^ inhibitory myeloid cells, and was associated with the failure of ICB therapy in mice.^21^ Notably, RIPK1-mediated resistance to therapy relies on its ubiquitin scaffold function rather than its kinase activity. However, other studies demonstrate ROS levels induced by TNF or some chemotherapeutic agents have been shown to promote the oligomerisation of RIPK1 and the RIPK1/RIPK3 interaction through the autophosphorylation of RIPK1 at Ser161.^44^ Therefore, chemotherapy-induced cancer cell death is also dependent on the kinase function of RIPK1, and the loss of RIPK1 function is likely to lead to increased drug resistance in cancer cells. Upregulation of RIPK1 in human melanoma cells after tunicamycin or thapsigargin treatment reduces the sensitivity of cancer cells to drug-mediated ER stress.^57^

Beyond chemotherapy, RIPK1 mediates resistance to targeted and ICB therapies *via* post-translational modifications and signalling reprogramming. In renal cell carcinoma (RCC), O-GlcNAcylation of RDA induces sunitinib resistance.^68^ For ICB resistance, interferon-γ receptor (IFNGR) signalling drives RIPK1 overexpression in tumour cells; genetic ablation of RIPK1 enhances ICB sensitivity in murine melanoma and breast cancer models.^92^^,^^93^ Clinical analyses further confirm that RIPK1 gene amplification correlates significantly with shortened progression-free survival.^93^ Mechanistically, RIPK1 regulates TNF signalling through NF-κB, promoting secretion of immunosuppressive cytokines (e.g., IL-6, TGF-β), impairing T/NK cell infiltration, and recruiting ARG1^+^ immunosuppressive myeloid cells to establish an immune-excluded microenvironment.^21^^,^^91^ Scaffold-stabilised NF-κB survival signalling thereby confers resistance to tyrosine kinase inhibitors and ICB.^91^ In contrast, targeted degradation of the RIPK1 scaffold (e.g., *via* PROTAC degrader LD4172) unleashes necroptosis, augments CD8^+^ T cell infiltration, reduces Treg accumulation, and significantly potentiates anti-PD-1 efficacy.^94^ Although multiple studies indicate a significant correlation between upregulated RIPK1 expression following chemotherapy and drug-resistant phenotypes, establishing a definitive causal relationship remains challenging. RIPK1 induction may primarily represent an adaptive response to chemotherapeutic stress (e.g., genotoxic or ER stress), rather than directly functioning as a driver of resistance. Collectively, RIPK1 functions as a critical node integrating intrinsic and extrinsic pathways governing ICB responsiveness, positioning it as a promising target for combinatorial strategies to overcome therapeutic resistance.

## 5. RIPK1 related drugs in cancer therapy

The identification of RIPK1 kinase as a critical mediator of cell death and inflammation presents promising therapeutic strategies for the treatment of human diseases.^95^ Based on binding sites and conformational regulation within the KD, small-molecule RIPK1 inhibitors are classified into three types: Type I (ATP-competitive) inhibitors bind to the ATP-binding pocket (active site), stabilise the DFG-in active conformation, and directly inhibit kinase activity; Type II inhibitors target both the ATP pocket and the adjacent allosteric hydrophobic pocket (DLG-out pocket), stabilising the DFG-out inactive conformation to block kinase activation; Type III (non-ATP-competitive) inhibitors bind to distal allosteric hydrophobic sites, indirectly inducing the DFG-out conformation and suppressing ATP binding to limit kinase activity.^25^^,^^96^ Due to off-target effects caused by direct ATP competition, Type I inhibitors exhibit limited selectivity, thus current clinical-stage candidates are predominantly Type II/III inhibitors.^97^ Besides, the chemotypes of RIPK1 inhibitors include indole-hydantoin (e.g., Nec-1s),^96^^,^^98^ dihydropyrazole (e.g., GSK’963).^99^^,^^100^ Some RIPK1 inhibitors, like DNL747 and DNL748, have shown promise in Alzheimer’s disease and amyotrophic lateral sclerosis^101^ (Table 2). However, their clinical trials are ongoing, paving the way for potential future commercialisation.

**Table 2.** RIPK1 inhibitors and degraders with antitumor activity.

Type	Drug	Structure	*In vivo/vitro* model	Kinase IC_50_/EC_50_/DC_50_	*t*_1/2_	Efficacy	Ref.
**Kinase inhibitor**							
Type I	Nec-1	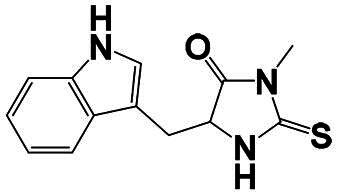	Colorectal cancer mouse	EC_50_: 182 nM	–	The number and size of macroscopical tumours ↓ The number of Ki-67 positive cells ↓	^103^
Type II	RIPK1-IN-7	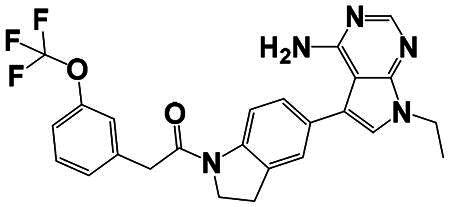	Melanoma mouse	IC_50_: 11 nM	2.254 h	Lung metastasis ↓ Necrotic cell death of human umbilical vein endothelial cell induced by tumour cells ↓	^105^
Type II	PK68	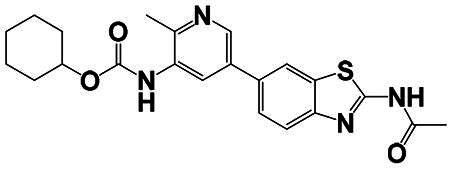	Melanoma mouse	IC_50_: 90 nM	1.3 h	Lung metastasis ↓	^104^
Type III	GNE684	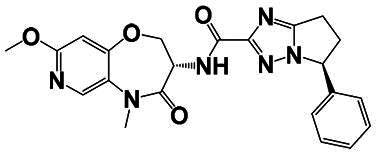	Mouse models of lymphoid malignancy (EL4) and myeloid malignancy (C1498)	IC_50_: 21 nM	–	Survival ↑	^123^
Type III	GSK’547	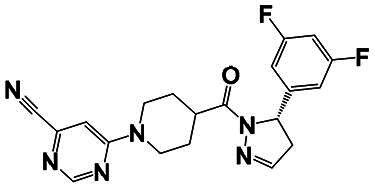	Pancreatic ductal adenocarcinoma mouse	IC_50_: 32 nM	–	Pancreatic tumour weights ↑ Survival ↑ T cell activation ↑ Liver metastases ↓	^106^
Type III	GSK3145095	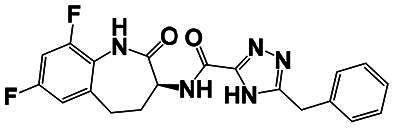	Pancreatic cancer mouse Pancreatic cancer patients	IC_50_: 6.3 nM	2.2 h	T-cell infiltration and activation ↑	^107^ ^,^ ^108^
**RIPK1 degrader**							
Type II+E3 ligase VHL	R1-ICR-5	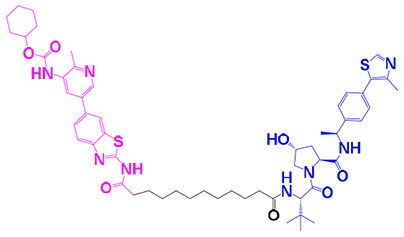	EO771 MCA-205 MC38 HT29 LIM1215 Breast cancer mouse	DC_50_: 0.1 ∼ 1 nM	–	Sensitivity of TNF-induced necroptosis ↑ Recruitment of activated TNFIFNy lymphocytes to the tumour microenvironment ↑ Survival ↑	^69^
Type II+E3 ligase VHL	LD4172	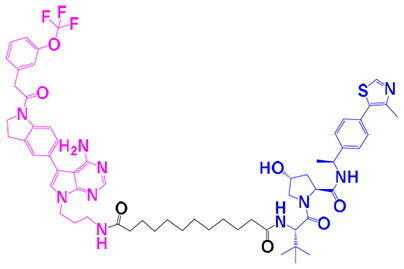	Melanoma mouse	DC_50_: 4 ∼ 400 nM	3.3 h	Sensitivity of tumours to anti-PD1 therapy ↑ Tumour-infiltrating lymphocyte responses ↑	^110^

Recent studies have demonstrated that the inhibition of RIPK1 kinase activity enhances antitumor immunity by modulating tumour-associated macrophages (TAMs), indicating that RIPK1 may serve as a promising immunomodulatory target for the development of innovative anticancer therapies.^102^ Nec-1 (Necrostatin-1), a type II inhibitor targeting the RIPK1 allosteric pocket, significantly reduces tumour burden (volume and number) in murine colon cancer models by inhibiting RIPK1 kinase activity and suppressing the JNK/c-Jun pathway.^103^ Jue *et al.* discovered PK68 as a novel inhibitor of RIPK1 kinase activity, suggested that PK68 can be used in the development of new therapies for the treatment of necroptosis-activated pathologies including inflammatory disorders and cancer metastasis.^104^ In melanoma models, PK68 and RIPK1-IN-7 potently inhibit pulmonary metastasis.^104^^,^^105^ Type III inhibitors GSK’547 and GSK3145095 enhance immunotherapy by promoting T-cell infiltration/activation in pancreatic ductal adenocarcinoma models, suppressing tumour growth and extending survival.^106^^,^^107^ Although preclinical studies demonstrate significant efficacy, the inherent disparities between murine models and human tumours, particularly in microenvironmental complexity, immune responses, and drug metabolism, necessitate cautious extrapolation to clinical settings. While murine systems remain valuable for mechanistic validation and conceptual exploration, their predictive accuracy for human therapeutic outcomes requires rigorous verification. Future efforts should prioritise more clinically relevant models, such as patient-derived organoids (PDOs) or patient-derived xenografts (PDXs), to enhance translational reliability. Notably, GSK3145095 successfully entered Phase II trials for antitumor therapy (NCT03681951), which planned to enrol approximately 220 participants over a 2-year duration.^108^ However, the trial was terminated prematurely due to undisclosed safety concerns, highlighting the challenges in the clinical development of RIPK1 inhibitors. The potential hepatotoxicity risk may stem from on-target effects due to high hepatic RIPK1 expression or off-target effects and metabolic properties of the compound itself. Future development must vigilantly monitor hepatotoxicity as a potential class effect and mitigate risks by optimising pharmacokinetic profiles, employing liver-targeted delivery strategies, or developing biomarkers to identify susceptible populations.

Based on existing studies, these apparent antitumor effects observed upon pharmacological inhibition of RIPK1 kinase activity may involve three interrelated pathways: (1) Inhibition of RIPK1 KD conformation may indirectly impair scaffold stability, thereby attenuating pro-tumorigenic signalling cascades (e.g., NF-κB-driven survival pathways); (2) Blockade of RIPK1-RIPK3-MLKL necrosome formation may promote caspase-8-mediated apoptosis, despite that RDA partially requires kinase activity; (3) Pharmacological inhibition of RIPK1 kinase activity may reprogram the necroptosis signalling cascade and potentiates antitumor immunity.^80^ However, the mechanism of ICD mediated by kinase activity requires the concomitant ablation of RIPK1 scaffold function. While type II/III kinase inhibitors suppress kinase activity, they paradoxically preserve the scaffold function, thereby limiting ICD induction and antitumor immunity. Furthermore, their suboptimal selectivity promotes off-target binding, driving hepatotoxicity and systemic inflammation. Consequently, enhancing target specificity and ablating the scaffolding function represent dual priorities for future drug development.

Compared to inhibitors, PROTAC-mediated RIPK1 degradation simultaneously ablates kinase activity and non-catalytic scaffolding functions, offering superior potential for reshaping the immune microenvironment and overcoming therapeutic resistance. PROTAC degraders employ heterobifunctional structures comprising a RIPK1-binding warhead (e.g., type II inhibitor PK68), an optimised linker (10-methylene chain), and an E3 ligase ligand (primarily VHL), which specifically induce RIPK1 ubiquitination and proteasomal degradation (Table 2). Degradation of RIPK1’s scaffold function eliminates its brake on RIPK3-MLKL signalling, leading to increased necroptosis and subsequent amplification of DAMP release (e.g., HMGB1, ATP), dendritic cell activation, and CD8^+^ T cell infiltration.^109^ When combined with radiotherapy or ICB, LD4172 increases anti-PD1 sensitivity by 300% in melanoma cancer models, while R1-ICR-5 with radiotherapy achieves 50% complete tumour regression and durable immune memory in TNBC.^69^^,^^110^ Acute degradation dysregulates TNFR1/TLR3/4 signalling, potentiating NF-κB/MAPK/IFN outputs and sensitising tumours to TNF/IFN, thereby reversing the cold tumour phenotype; concurrently, disruption of the TRADD-RIPK1 complex drives TRADD-dependent RIPK3 activation, promoting apoptosis while inhibiting pro-survival pathways (e.g., NF-κB pathways).^69^^,^^110^ Despite overcoming limitations of inhibitors, systemic degradation may compromise immune homeostasis (e.g., autoinflammation due to impaired K376 ubiquitination), and current degraders face suboptimal tissue distribution and pharmacokinetic constraints, necessitating toxicity assessment and structural optimisation to balance efficacy and safety.^93^ Interestingly, Xin Yu *et al.* developed LD4172, a highly potent and selective PROTAC-based RIPK1 degrader, which was designed to disrupt the scaffold function of RIPK1, thereby effectively inducing specific degradation of RIPK1 and enhancing the sensitivity of multiple preclinical tumour models to anti-PD-1 therapy.^110^

Despite its therapeutic potential through simultaneous degradation of both kinase and scaffold functions of RIPK1, the clinical translation of PROTAC technology faces several challenges: (1) Pharmacokinetics and tissue selectivity: The relatively large molecular size of PROTACs often results in suboptimal oral bioavailability, tissue permeability, and overall pharmacokinetic profiles. Systemic degradation of RIPK1 may disrupt its physiological functions in normal tissues, leading to on-target toxicities such as immune homeostasis imbalance. Emerging strategies (e.g., antibody-PROTAC conjugates and cell-type-specific ligand-modified PROTACs) aiming to achieve tumour-selective degradation represent promising future directions. (2) Long-term safety unknown: The immunological consequences and potential toxicities associated with chronic, systemic RIPK1 degradation remain inadequately characterised, necessitating careful monitoring in clinical studies. (3) Drug resistance: Tumour cells may develop resistance to PROTACs *via* downregulation of E3 ligases or mutations in the target protein. Therefore, advancing PROTACs towards clinical application requires thorough evaluation of their toxicity profiles and the development of tissue-specific degradation strategies.

## 6. Conclusions and prospects

RIPK1 serves as a central hub regulating cell death and immunity, exerting dual roles in tumour progression through a dynamic balance between its kinase activity and scaffold function: (1) its scaffold-dependent function stabilises Complex I to activate the NF-κB pathway, driving pro-survival gene expression and fostering an immunosuppressive microenvironment that promotes therapy resistance and metastasis; (2) its kinase-dependent activity mediates necrosome assembly and induces ICD, thereby reversing the cold tumour phenotype, though this effect is markedly attenuated by its scaffolding action. This functional duality underscores the complexity of targeting RIPK1, necessitating intervention strategies tailored to tumour type, death pathway status, and individualised immune microenvironment features. Current therapeutic strategies primarily rely on type II/III kinase inhibitors and degraders. The anti-tumour effects of kinase inhibitors are not achieved through direct ICD induction, but rather through inhibiting kinase-mediated pro-survival signalling, reprogramming tumour cell death modalities, and reshaping the immune microenvironment in specific tumour contexts. While RIPK1 inhibitors selectively suppress kinase activity, they fail to eliminate scaffold function, leading to persistent pro-survival signals (e.g., NF-κB) and limited ICD efficacy. In contrast, RIPK1 degraders employ the ubiquitin-proteasome system to completely clear RIPK1, simultaneously ablating scaffold-mediated survival signals and relieving inhibition on the RIPK3-MLKL pathway, significantly enhancing anti-tumour immunity.

Therapeutic targeting of RIPK1 through degradation to enhance antitumor immunity entails potential risks, such as hepatotoxicity or autoinflammation, due to its critical role in maintaining immune homeostasis in normal tissues, especially in the liver and intestine. This situation emphasises the pressing need to develop tumour-specific delivery systems or conditionally activated degraders. The success of future clinical trials will depend on prospective, biomarker-driven strategy. RIPK1-targeted therapy may depend not only on the drug itself but also on precise patient selection strategies. Future clinical studies are necessary to explore predictive biomarkers of efficacy and toxicity, such as the expression and activation levels of tumour RIPK1 (e.g., detected by p-RIPK1 immunohistochemistry), immune infiltration characteristics (e.g., CD8+ T cell density, immune exclusion phenotype), and specific genomic signatures (e.g., NF-κB pathway activation, specific oncogenic mutations). By doing so, patient populations most likely to benefit can be identified while avoiding severe toxicity in susceptible individuals (e.g., those with liver dysfunction), thus increasing the success rate of clinical trials and uncovering the true clinical value of RIPK1 targeting.

Future research should further explore RIPK1 within specific cancer contexts, such as RCC. The potential crosstalk between its core driver pathway mTOR and RIPK1 signalling remains underexplored and may offer novel targets to overcome therapy resistance. Meanwhile, integrating RIPK1 status with RCC multi-omics data (e.g., metabolomics, immune profiling) using machine learning could refine prognostic models and patient stratification strategies.^111^ Additionally, given the close relationship between mitochondrial dysfunction and necroptosis, assessing metrics like mtDNA copy number may serve as a valuable biomarker for predicting responses to RIPK1-targeted therapy.

From the perspective of drug development, progress in PROTAC technology and nano-delivery systems can transform first-generation degraders into intelligent, tissue-specific, and multi-targeting agents. This transformation enables precise control of specificity, overcomes off-target toxicity, and addresses compensatory resistance. Moreover, as RIPK1 function is significantly regulated by PTMs such as ubiquitination and phosphorylation, selectively targeting these PTMs may provide an alternative therapeutic approach. In terms of clinical strategies, combining RIPK1 degraders with ICB takes advantage of a potent synergy between immune suppression reversal and anti-tumour immune activation. Pharmacological degradation of RIPK1 remodels the TME and simultaneously induces ICD, thereby effectively reversing the cold tumour phenotype and sensitising tumours to anti-PD-1 therapy. This synergy is evidenced by increased infiltration of CD8^+^ T cells, a decrease in immunosuppressive myeloid cells, and an enhanced secretion of immunostimulatory cytokines such as IFN-γ and IL-2. This combinatorial approach can be extended beyond ICB. For example, the combination of RIPK1 targeting and CD40 agonists could theoretically act synergistically to further reverse immunosuppression and induce ICD, representing a promising new direction for combination immunotherapy. Moreover, RIPK1 degradation also offers a compelling strategy for synergizing with radiotherapy. Radiotherapy triggers the release of cytokines such as TNF and IFN. Inhibition of RIPK1 significantly heightens the sensitivity of tumour cells to these signals. This promotes various cell death mechanisms, including apoptosis and necroptosis, thus amplifying the immunostimulatory effect of radiotherapy and potentially enhancing abscopal effects.

In conclusion, translating RIPK1-targeted therapy from preclinical research to clinical practice necessitates transforming complex biological understandings into precise clinical instruments, utilising innovative drug technologies to reduce toxicity, and implementing biomarker-guided patient stratification to guarantee the success of clinical trials. This ultimately provides new solutions for patients who are resistant to immunotherapy.

## Data Availability

Data sharing is not applicable to this article as no new data were created or analysed in this study.

## Associated data

This section collects any data citations, data availability statements, or supplementary materials included in this article.

## Abbreviations

RIPK1: Receptor-interacting serine/threonine-protein kinase 1
TME: tumour microenvironment
PCD: programmed cell death
ER: endoplasmic reticulum
MOMP: mitochondrial outer membrane permeability
ICD: immunogenic cell death
DAMPs: damage-associated molecular patterns
ICB: immune checkpoint blockade
RIP: Receptor-interacting protein
KD: kinase domain
ID: intermediate domain
RHIM: RIP homotypic interaction motif
DD: death domain
PTMs: post-translational modifications
IKK: IκB kinase
RDA: RIPK1-mediated apoptosis
MLKL: mixed lineage kinase domain-like protein
PIP: phosphatidylinositol phosphate
TNBC: triple-negative breast cancer
DCs: dendritic cells
RCC: renal cell carcinoma
IFNGR: interferon-γ receptor
GSDMD: gasdermin D
HMGB1: high mobility group box-1 protein
ATP: adenosine triphosphate
